# Preparation and Application of Carbon Dots Nanozymes

**DOI:** 10.3390/antiox13050535

**Published:** 2024-04-27

**Authors:** Jichuan Kong, Feng Zhou

**Affiliations:** School of Medicine, Henan Polytechnic University, Jiaozuo 454000, China; 15514778874@163.com

**Keywords:** CDs, nanozymes, synthesis, application, enzyme-like activity

## Abstract

Carbon dot (CD) nanozymes have enzyme-like activity. Compared with natural enzymes, CD nanozymes offer several advantages, including simple preparation, easy preservation, good stability and recycling, which has made them a popular research topic in various fields. In recent years, researchers have prepared a variety of CD nanozymes for biosensing detection, medicine and tumor therapy, and many of them are based on oxidative stress regulation and reactive oxygen species clearance. Particularly to expand their potential applications, elemental doping has been utilized to enhance the catalytic capabilities and other properties of CD nanozymes. This review discusses the prevalent techniques utilized in the synthesis of CD nanozymes and presents the diverse applications of CD nanozymes based on their doping characteristics. Finally, the challenges encountered in the current utilization of CD nanozymes are presented. The latest research progress of synthesis, application and the challenges outlined in the review can help and encourage the researchers for the future research on preparation, application and other related researches of CD nanozymes.

## 1. Introduction

Enzymes, as a kind of biocatalyst with high efficient catalytic ability to catalyze various biochemical reactions specifically, are widely used in the field of medicine and industrial production, etc. [1,2,3]. The vast majority of natural enzymes are composed of proteins, and only a small number of them are ribozymes composed of RNA, all of which have highly efficient and specific catalytic properties [4,5]. The catalytic ability of enzymes will be affected by the reaction conditions; in the optimal temperature, pH and other reaction conditions, the enzyme can exert the maximum catalytic ability. On the contrary, the enzyme activity is reduced, and in extreme conditions it can even lead to the loss of enzyme activity [6]. The poor stability of natural enzymes, the expensive cost of preparation and preservation, the sensitivity to extreme reaction conditions and the difficulty of recovery and recycling all limit the application of natural enzymes in practice [7,8,9,10,11]. Nanomaterials based artificial mimicking enzymes, also named as nanozymes, have attracted considerable attention due to their higher stability and lower cost than that of natural enzymes [12,13,14,15,16]. In recent years, nanozymes have been used in biomedical applications, including diabetes treatment, oral infection treatment, anti-aging and tumor therapy, due to their excellent properties, including catalytic activities [17,18,19,20,21]. The preparation of nanozymes with enzyme-like activity based on carbon nanomaterials has attracted great interest from researchers [22,23]. The enzyme-like activity of nanozymes is affected by various factors, including structural features and the external environment [24]. There are many types of nanozymes based on carbon nanomaterials, including CDs, graphene, fullerenes and carbon nanorods, etc.

CDs have a small size of less than 10 nm, resulting in a large surface area and an increased abundance of surface groups, and the existence of carbon-rich, nitrogen-rich and oxygen-rich groups on the surface of CDs has been proposed [25,26,27]. Graphene CDs, carbon quantum dots, carbon nanodots and carbonized polymer dots are the four most common classifications of CDs, and different types of CDs have different structures and different functions [28]. Due to their small size, CDs can easily cross the cell membrane into the cytoplasm, and then into other organelles for action [29]. CDs can also cross structures that protect the organism from aggression, such as the blood–brain barrier, and different sizes, surface modifications and environments can lead to altered entry mechanisms [30]. CDs have unique fluorescent properties due to surface conjugation and other reasons and are a relatively new type of nanomaterial with better stability compared to other organic molecular dyes [31,32,33]. In addition, CDs also play a role in fighting against COVID-19 and other antiviral diseases [34]. CDs have also been applied in biomedicine [35]. CD nanozymes, compared to other materials, have better biocompatibility, lower toxicity and excellent stability and have been widely used in medicine, bio-detection and disease treatment, becoming a hot topic in current research [36,37,38,39].

CD nanozymes have strong mimetic enzyme activities and the proposed enzyme-like activities of CD nanozymes mainly include oxidase-like (OXD-like), peroxidase-like (POD-like), superoxide dismutase-like (SOD-like) and oxidoreductase-like activities [40,41]. The enzyme-like activities of CD nanozymes also exhibit catalytic properties similar to natural enzymes, such as how peroxidase-like enzymes of CDs were able to rapidly convert 3,3′,5,5′-tetramethylbenzidine (TMB) into colored products in the presence of hydrogen peroxide (H_2_O_2_), generating a large amount of reactive oxygen species (ROS) such as hydroxyl radicals (•OH), which can be used for antimicrobial therapy [42,43,44]. Compared with natural enzymes, CD nanozymes have more stable enzyme-like activity and photocatalytic properties. Surface modification, size and shape, and doping elements can also be used to further enhance the enzyme-like activity and other properties of CD nanozymes [45,46,47]. However, in the case of doping metal ions, the optical properties of the CD nanozymes may also be affected, which may lead to the decrease in the fluorescence quantum yield and other situations [48]. The smaller-sized CD nanozymes can be better dispersed in the solvent and promote the reaction in the solvent [49]. CD nanozymes can also be combined with other materials to form composites, which can inherit and improve the catalytic ability and other related properties of CD nanozymes, making the applications of CD nanozymes more extensive [50,51,52].

**Figure 1:**
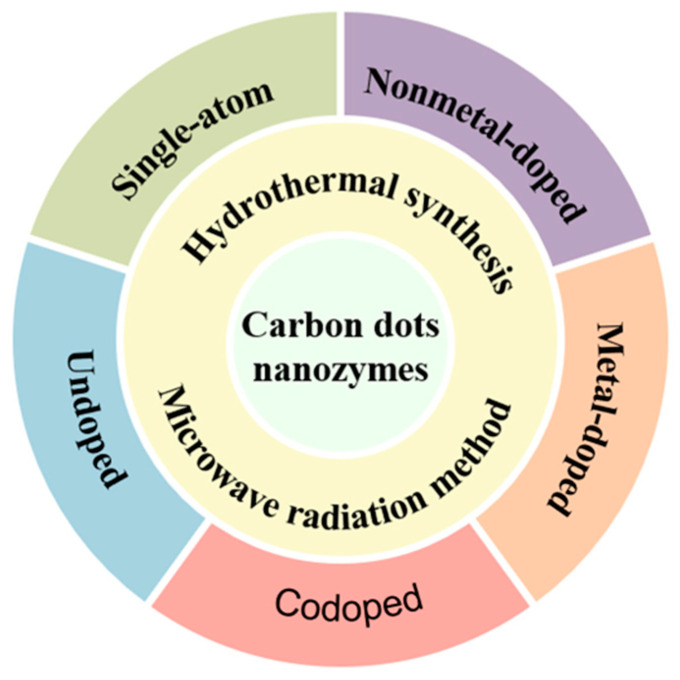
Preparation of CD nanozymes and their doping types.

This review mainly introduces the main preparation methods of CD nanozymes, hoping to help understand the preparation methods of CD nanozymes and provide ideas for the design and development of CD nanozymes in the future. In addition, the applications of CD nanozymes are also introduced based on different doping types, so as to understand the direction of different doped CD nanozymes in practical applications and to achieve the precise design and applications of CD nanozymes(Figure 1).

## 2. Preparation of CDs Nanozymes

Currently, the preparation of CDs can be divided into two strategies: one is the “top-down” method; the other is the “bottom-up” method [53,54]. The top-down method mainly involves decomposing or cutting the larger carbon materials through physical and chemical methods to obtain carbon nanoparticles, and the sizes of the obtained carbon nanoparticles vary, which refer to electrochemical techniques, laser ablation techniques, etc. The bottom-up method is mainly to obtain the CDs by fusing the molecular precursors or other polymerization precursors, and the CDs with controllable sizes and rich carbon sources can be obtained by this method, and the main methods include hydrothermal synthesis techniques and microwave radiation techniques [55,56,57]. The top-down method for synthesizing CDs requires strict reaction conditions, longer reaction time, lower efficiency and more by-products. The “bottom-up” method can control the size of CDs, making the materials easy to obtain and the reaction operation simple [58,59,60]. Currently, the preparation of CDs is mainly based on the bottom-up strategy, mainly obtaining the desired CDs through hydrothermal synthesis or microwave radiation [61]. The same synthesis strategy can be used for CD nanozymes. Due to the advantages of the bottom-up approach, this article mainly reviews the preparation of CD nanozymes using this strategy. Table 1 summarizes the recent studies on the preparation of CD nanozymes and related enzyme-like activity by the “bottom-up” method.

### 2.1. Hydrothermal Synthesis

Hydrothermal synthesis is the most commonly used method for the preparation of CD nanozymes. The general procedure of hydrothermal synthesis is to dissolve the molecular precursors or other polymerization precursors in ultrapure water or other solvents to form a solution, transfer it to a reactor, react under high temperature and pressure for a period of time, cool the mixture to room temperature, and then purify it by column chromatography to obtain the prepared carbon-dot nanozymes. Hydrothermal synthesis is relatively simple, low-cost and environmentally friendly and non-toxic compared with other preparation methods. Moreover, the optical properties of CDs obtained by the hydrothermal method are excellent, and the post-processing operation is convenient and fast [83,84,85]. However, the reaction time and temperature have a direct influence on the properties and functions of the prepared CDs, so the reaction time and temperature should be strictly controlled during the reaction process [86].

**Figure 2:**
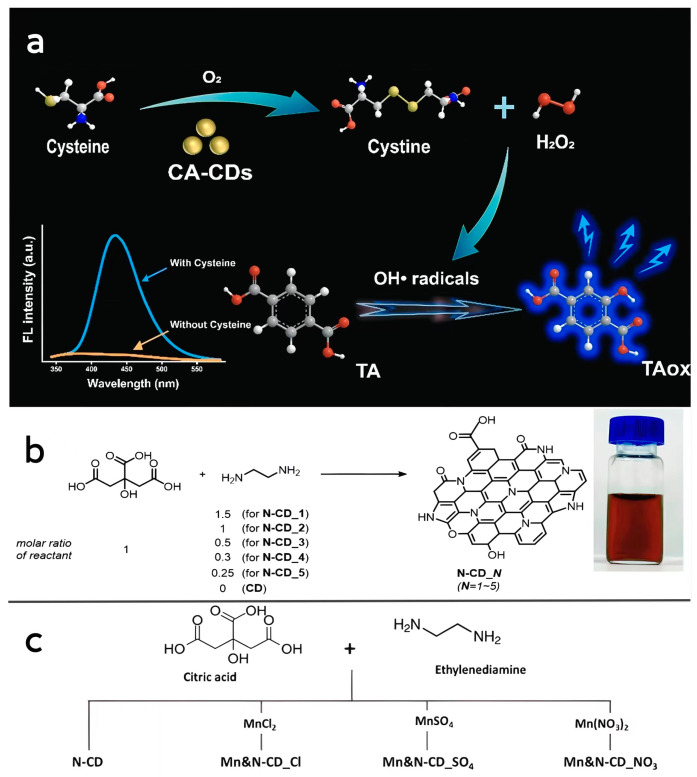
(**a**) Schematic representation of cysteine-like oxidase activity of CA-CDs. Reprinted with permission from ref. [87]. Copyright 2022, Elsevier. (**b**) Photographs of the preparation process of N-CDs and the prepared N-CD samples suspended in water. Reprinted with permission from ref. [64]. Copyright 2021, Royal Society of Chemistry. (**c**) Schematic diagram of the preparation process of Mn, N-CDs. Reprinted with permission from ref. [88]. Copyright 2023, Royal Society of Chemistry.

Lin et al. [87] dissolved citric acid in water and reacted it at 200 °C for 12 h. Undoped citric acid CD nanozymes (CA-CDs) were obtained after dialysis purification. The obtained CA-CDs were uniformly distributed with an average particle size of 1.65 nm, and Fourier-transform infrared analysis (FTIR) revealed that C=C double bonds were formed on their surfaces, and carbon-13 nuclear magnetic resonance (13C-NMR) analysis showed that the newly synthesized CA-CDs contained aromatic structures. The prepared CA-CDs have cysteine oxidase-like activity, which can catalyze the oxidative decomposition of cysteine to obtain cystine and H_2_O_2_, and the prepared CA-CDs have a higher affinity for cysteine compared to natural cysteine oxidase (Figure 2a).

Lee et al. [64] used citric acid and ethylenediamine as precursors to obtain N-CDs with peroxidase-like activity by reacting the formulated solvents at a high pressure of 200 °C for 1 h, cooling to 25 °C and purifying by column chromatography (Figure 2b). Due to the different ratios of carbon and nitrogen sources used in the reaction, the size of all types of synthesized CD nanozymes ranges from 3.4 to 17.8 nm. By comparing the optical properties of CD nanozymes with different ratios of carbon and nitrogen sources, it was found that the N-doping content of the CD nanozymes played a key role in influencing their optical properties. Compared with the undoped CD nanozymes, the N-CDs can oxidize TMB more rapidly, and the maximum enzymatic reaction velocity (Vmax) was also higher than that of the undoped CD nanozymes in all cases. It is noteworthy that the value of Km, the affinity between the enzyme and the substrate, changes only slightly with the change in the N-doped content, which also suggests that doping of heteroatoms plays an important optimizing role on the enzymatic activity of CD nanozymes. Kang et al. [88] also obtained Mn, N-codoped CD nanozymes by hydrothermal synthesis with the addition of a manganese source, and the codoped CD nanozymes had higher Vmax and lower Km values than N-CDs, showed higher affinity for the substrate and possessed a more excellent peroxidase-like catalytic activity, which also indicates that metal doping has a significant enhancement effect on the activity of CD nanozymes. This also indicates that metal doping has a significant enhancement effect on the activity of CD nanozymes, but the related mechanism has not been thoroughly studied yet (Figure 2c).

For comparison, Zhuo et al. [68] used citric acid and ethylenediamine as precursors to synthesize Mn-CDs and bare CDs through hydrothermal synthesis and also prepared pure Mn samples for comparison. The preparation process involved dissolving citric acid, ethylenediamine and manganese sources in ultrapure water, reacting the obtained mixture in an autoclave at 180 °C for 10 h and purifying the supernatant by centrifugation followed by dialysis for 24 h to obtain the prepared Mn-CDs. The synthesized Mn-CDs possessed oxidase-like activities and the oxidase-like activities possessed were higher than those of the CD nanozymes synthesized using only citric acid or ethylenediamine as a single precursor. The possible reason is due to the synergistic effect of Mn doping on the CDs, which enhances the enzyme activity.

The solvent pyrolysis and hydrothermal synthesis methods for the preparation of CD nanozymes are not very different in nature, and thus both can be categorized as hydrothermal synthesis methods for the preparation of CD nanozymes.

### 2.2. Microwave Radiation Method

The microwave radiation method is a green and convenient method to obtain CD nanozymes, through which CD nanozymes containing dopant elements can be quickly and efficiently obtained from the prepared organic mixtures [55]. The microwave radiation method is faster compared to the hydrothermal method for the preparation of CD nanozymes, but the morphological regularity of the prepared CD nanozymes is poor [89]. The specific procedure is to react the precursor mixture in a microwave synthesis reactor at a certain power and temperature, followed by cooling, purifying, and removing impurities through dialysis and other methods to obtain the pure CD nanozymes. The CD nanozymes obtained by the microwave radiation method can enhance their fluorescence properties by surface passivation or purification and decontamination to improve the breadth of their application in practical applications.

**Figure 3:**
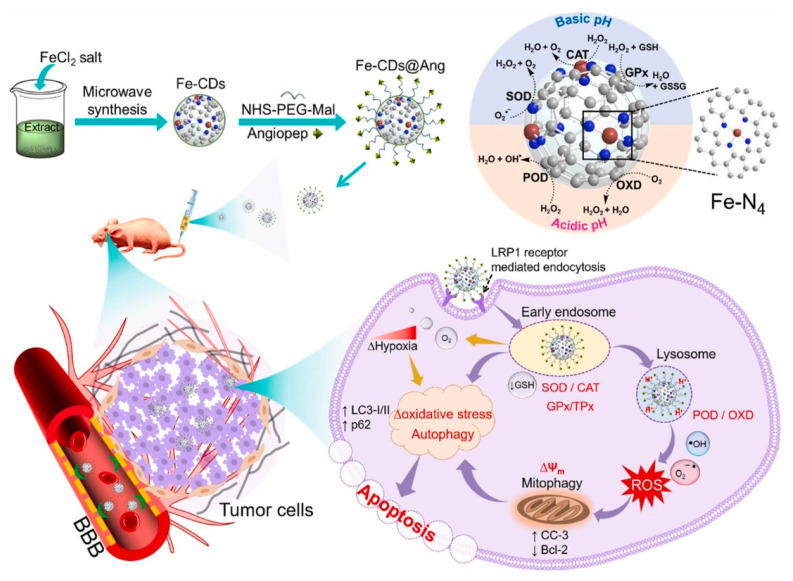
Schematic illustration of the preparation of Fe-CD nanozyme and the enzymatic cascade initiated by angiopep-2-modified Fe-CD nanozyme for ROS regulation to induce the autophagy–lysosome pathway for GBM therapy. Reprinted with permission from ref. [80]. Copyright 2022, Elsevier.

Muhammad et al. [80] used the leaves of bougainvillea plants as a carbon source precursor material for the synthesis of CD nanozymes. The leaves were pulverized and a mixture of 10 g of pulverized leaves with 100 mL of ethanol-water solute on miscible at 1:1 ratio was configured, after which ferric chloride was added as a dopant element, and the obtained mixture was processed in a 630 W microwave oven to obtain the different sizes of the Fe-CDs, which were further purified by centrifugation and other steps to obtain ultra-small CD-loaded iron monoatomic nanozymes (Figure 3). It was found that Fe-CDs have oxidase-like, catalase-like, superoxide dismutase-like, peroxidase-like, glutathione peroxidase-like and thiol peroxidase-like activities and thus could be used to eliminate the damage caused by reactive oxygen species generated in the body to keep the organism in a relatively stable state. By comparing Fe-CDs with non-metal doped N-CDs, it could be observed that Fe-CDs had relatively high oxidase-like, peroxidase-like and superoxide dismutase-like activities, but their catalase-like activities were significantly lower compared to N-CDs. N-CDs exhibited almost nonexistent glutathione peroxidase-like and thiol peroxidase-like activities, which also reflected the influence that different elemental dopings have on CD nanozymes’ activity.

Bijalwan et al. [81] prepared metal-doped Cu-CD nanozymes and Mn-CD nanozymes, as well as undoped CD nanozymes, through microwave radiation solid-phase synthesis. For the preparation of Cu-CDs, 0.75 g of urea, 0.25 g of citric acid and 0.03 g of copper acetate were used as carbon, nitrogen and dopant element precursors, respectively, the mixtures were pulverized and placed in a microwave reactor at 220 °C for 10 min, then cooled down to room temperature, purified by centrifugation and decontamination, and dried to obtain the Cu-doped CD nanozymes. At the same time, the same method was used to obtain the Mn-CD nanozymes with manganese tetrahydrate as a manganese precursor. Mn-doped CD nanozymes with manganese acetate as a manganese precursor and undoped CD nanozymes with urea and citric acid as precursors were also obtained by the same method. It is worth noting that solid-phase synthesis was used for the synthesis of CD nanozymes by this method, which eliminated unnecessary solvents and made the preparation of CD nanozymes more convenient and efficient compared with the previous synthesis methods. The three prepared CD nanozymes were all around 2–15 nm in size, and only the Cu-doped CD nanozymes possessed peroxidase-like activity in a neutral pH environment, and with the change in pH, the enzyme activity showed different degrees of changes, with the highest enzyme activity in an acidic environment at pH 4 and the lowest enzyme activity in an alkaline environment at pH 9, and so it was hypothesized that their peroxidase-like activity might result from the synergistic interactions between copper and CDs as well as the interaction of radical cations with charge transfer complexes. As the enzyme can function over a wide range of pHs, it can be used as a peroxidase-like mimetic nanozyme under physiological conditions.

## 3. Applications of CDs Nanozymes

The application of CD nanozymes was explored according to different doping types, as different doping elements during the preparation process may influence the properties of CD nanozymes. CD nanozymes were widely used in biosensing, crop growth, detection and therapy due to their unique physical properties and advantages, including having catalytic properties that mimic those of natural enzymes [76,90,91,92].

### 3.1. Single-Atom Nanozymes

Single-atom nanozymes stand out from conventional nanomaterials due to their unique geometrical and electronic structures, providing them with distinctive advantages. Unlike ordinary nanomaterials, single-atom nanozymes offer benefits such as a uniform distribution of active sites and minimal interference among individual atoms. These characteristics enable single-atom nanozymes to be effectively dispersed in solvents, enhancing their overall performance. These factors contribute to the widespread utilization of single-atom nanozymes across multiple sectors [93,94,95]. As a result of their enhanced properties, single-atom nanozymes exhibit superior performance and find applications in various fields, such as biomedicine. The properties exhibited by single-atom nanozymes vary in size, shape, temperature and pH [96]. Moreover, the higher efficiency of metal utilization in single-atom nanozymes contributes to reducing preparation costs [97,98].

**Figure 4:**
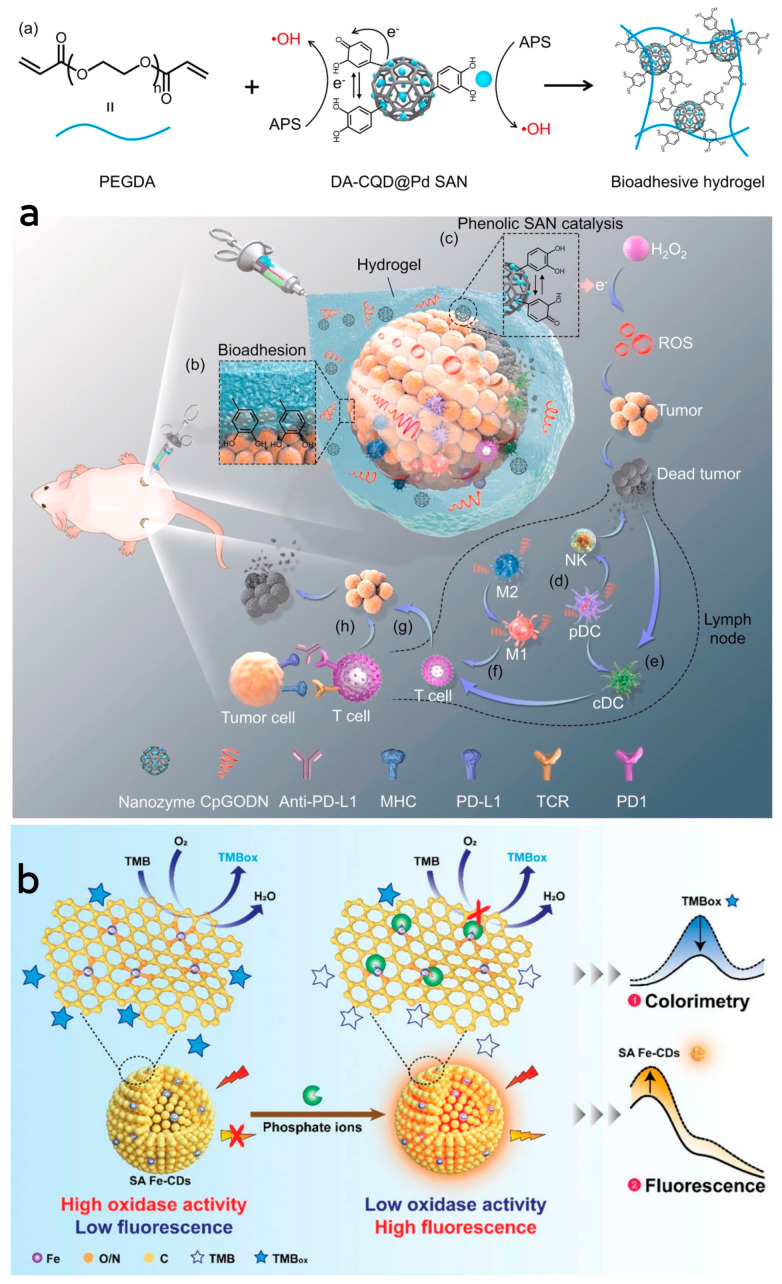
(**a**) Schematic illustration of Pt single−atom nanozyme complexes combined with CpGODN hydrogels for catalytic immunotherapy of tumors. Reprinted with permission from ref. [99]. Copyright 2021, Elsevier. (**b**) Schematic illustration of the dual-mode colorimetric and fluorometric sensing of Pi based on SA Fe-CDs. Reprinted with permission from ref. [101]. Copyright 2022, Wiley-VCH GmbH.

He et al. [99] synthesized phenolic platinum single-atom composite CD nanozymes by the hydrothermal synthesis method and subsequent treatment. This nanozyme exhibited better peroxidase-like and persulfatase-like activities, capable of catalyzing the generation of ROS from H_2_O_2_ in the tumor microenvironment, leading to the destruction of tumor cells. Subsequently, a bioadhesive hydrogel containing DA-CQD@Pd single-atom nanozymes and CpGODN was formed through a free radical polymerization reaction catalyzed by the single-atom nanozymes. Upon injection, this hydrogel formed a stable adhesion around the tumor, providing a localized site for immune response activation. The controlled release of CpGODN from the hydrogel sustained immune system activation, minimizing systemic exposure, reducing toxicity, and protecting CpGODN from degradation. This combined approach, along with the immune checkpoint inhibitor anti-PD-L1, enabled local immunomodulation, maximizing therapeutic efficacy and preventing tumor metastasis through catalytic immunotherapy. Dendritic cells take up CpGODN from the hydrogel, activating them to trigger a natural immune response by secreting interferon-alpha. Simultaneously, CpGODN induces polarization of M2 to M1 macrophages, facilitating the activation of antigen-presenting cells and T-cells, thereby initiating an adaptive immune response. The synergistic action of the hydrogel and anti-PD-L1 therapy significantly hindered primary and distal tumor growth, increased survival rates, and enhanced systemic anti-tumor immune responses. (Figure 4a). For the first time, Zhang et al. [100] prepared Pt single-atom nanozymes assembled with dual enzymatic peroxidase-like and glutathione oxidase activities. These assemblies synergize enzyme activity, photothermal effect and chemotherapeutic activity to inhibit the growth of tumor cells. Furthermore, they exhibit better biocompatibility, thus reducing damage to the organism’s cells. The enzyme makes better use of GSH to prevent the depletion of generated ^1^O_2_ and shows photothermal therapeutic effects at low temperatures. It also enables sustained drug release through depolymerization and drug release controlled by the tumor microenvironment or laser. Additionally, the enzyme assembly effectively regulates the intracellular redox balance, achieving highly efficient tumor growth inhibition.

Li et al. [101] synthesized a small-sized iron monoatomic CD nanozyme with a more stable oxidase-like activity (Figure 4b). Various characterization techniques revealed that iron atoms were uniformly dispersed as single atoms in the carbon matrix, forming abundant Fe-O/N active centers. Monatomic iron in the Fe-O/N active centers triggered the decomposition of dissociated O_2_ and generated highly reactive superoxide radicals which rapidly oxidized the TMB to its blue oxidation state (oxTMB). Phosphate ions (Pi) competitively inhibited the oxidase-like activity of the Fe-monatomic nano-enzymes because the adsorbed Pi occupied the catalytically active site. The sensitive and selective detection of Pi can be achieved through variations in UV-vis spectra and fluorescence spectra of the single-atomic iron-doped carbon dots. Muhammad et al. [80] developed ultra-small CD-loaded iron monoatomic nanozymes with multi-species enzymatic activity using the microwave radiation method. The CDs demonstrated OXD-like activity under physiological pH and temperature conditions. Remarkably, the enzyme exhibited enhanced POD activity in acidic environments, intensifying oxidative stress levels in tumor cells. The iron monoatomic nanozymes could convert superoxide anions into oxygen and H_2_O_2_, thereby regulating intracellular ROS balance. Moreover, these nanozymes accumulated in acidic lysosomes displayed OXD- and POD-like activities that disrupted lysosomal degradation and activated autophagy. By triggering autophagy, the iron monoatomic nanozymes facilitated the breakdown of damaged organelles and proteins within tumor cells, ultimately leading to cell death. Furthermore, the nanozymes were capable of modulating ROS levels in the tumor microenvironment, inhibiting tumor growth and incorporating peptides for BBB penetration through surface modification, effectively targeting GBM cells to enhance therapeutic efficacy. Notably, the enzyme combatted drug resistance in solid GBM tumors by engaging the ROS-mediated autophagy signaling pathway. Their GPx- and TPx-like activities enabled the regulation of ROS levels, consequently impacting the redox balance of tumor cells and promoting cell death to achieve therapeutic outcomes, establishing them as a promising novel nanodrug (Figure 3). Han et al. [102] introduced phenanthroline as a mediating ligand to synthesize ultra-small CD-loaded Fe monoatomic nanozymes by the hydrothermal method. The enzyme exhibited a higher degree of graphitization compared to conventionally synthesized Fe monoatomic nanozymes, resulting in enhanced electron transfer efficiency. This suggests a higher peroxidase activity within this enzyme class, facilitating the catalysis of enzymatic reactions. Analogous to metalloenzymes in organisms, isolated iron atoms present on the enzyme’s surface served as active centers, promoting the catalysis of H_2_O_2_ decomposition to produce highly toxic •OH, ultimately leading to cancer cell-specific apoptosis. The enzyme’s ultra-small size (3–5 nm) enabled it to effectively penetrate deep into tumor tissue, where it efficiently eradicated tumor cells. Additionally, when exposed to near-infrared light, the enzyme could convert light energy into heat energy, which synergistically complemented chemodynamic therapy (CDT) in inhibiting the growth of tumor cells.

Furthermore, single-atom nanozymes like Ir [103], Mn [104,105,106] and Co [107,108] are utilized for biomedical and environmental improvements, offering significant advantages over other nanomaterials.

### 3.2. Nonmetal-Doped CD Nanozymes

**Figure 5:**
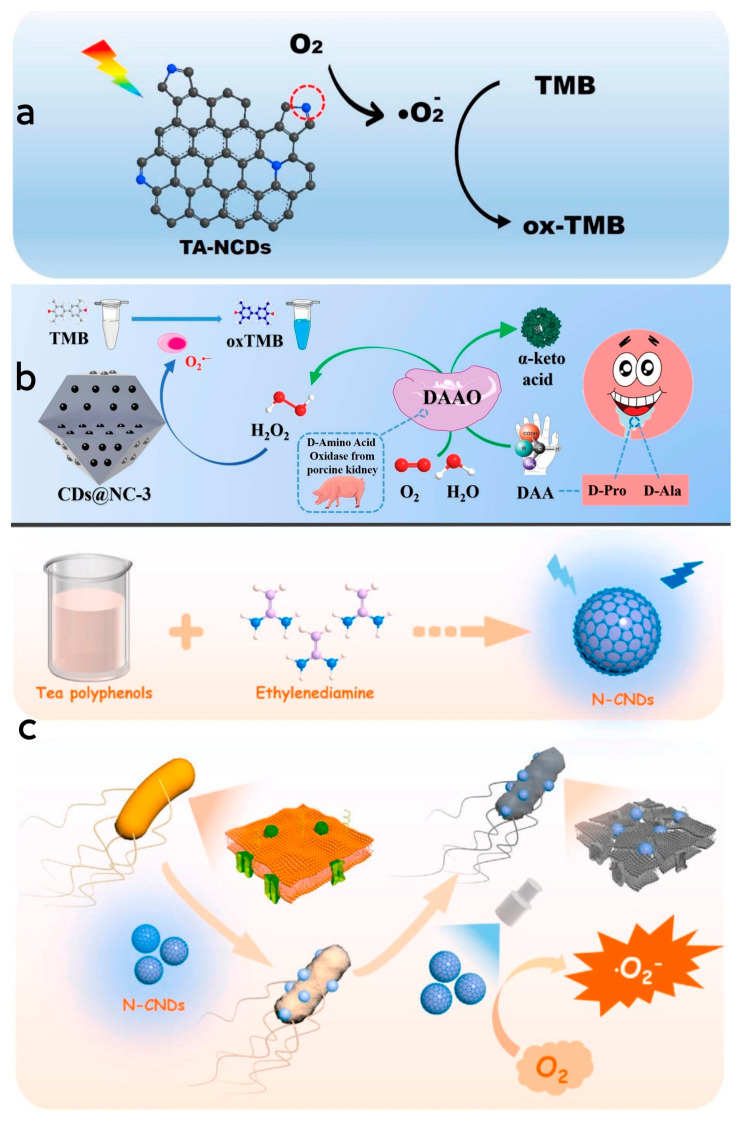
(**a**) Schematic diagram of the mechanism of TMB catalyzed by N-CDs. Reprinted with permission from ref. [66]. Copyright 2021, Elsevier. (**b**) Schematic diagram of detection mechanism of D-Pro and D-Ala by using CDs@NC-3. Reprinted with permission from ref. [113]. Copyright 2021, Elsevier. (**c**) Schematic diagram of the preparation process of N-CDs and their mechanism in the sterilization process. Reprinted with permission from ref. [65]. Copyright 2023, Elsevier.

In the preparation process of CDs, doped CDs can be modified by incorporating a small quantity of dopant atoms, which serves to enhance their quantum yield and alter their physical and chemical properties [109]. By doping elements into CDs during their preparation, the resulting CDs exhibit improved fluorescence performance and catalytic capability when contrasted with CDs produced via conventional techniques, thereby broadening their range of applications [110]. Moreover, the introduction of doping elements is pivotal in regulating the properties and catalytic performance of mimetic enzymes, while non-metallic doping exhibits superior biocompatibility with organisms and causes less cellular damage, making them more suitable for applications in biomedicine or therapeutics [111,112]. Through hydrothermal synthesis, Wang et al. [66] successfully synthesized a series of nitrogen-doped CD nanozymes with various molar ratios of precursor substances. These nanozymes exhibited oxidase-like activity, wherein the pyrrolyl nitrogen serves as both the catalytically active site and the substrate-binding site. The oxidation of TMB was catalyzed by activating oxygen to form superoxide radicals. The generation of reactive oxygen species was attributed to the electrons being excited into the conduction band under light stimulation to facilitate the reduction in oxygen. Remarkably, under visible light irradiation, the enzyme demonstrated strong binding to specific targets in mitochondria, leading to its enrichment in the mitochondria of HeLa cells. This localization resulted in the killing of nearly 60% of HeLa cells, underscoring the enzyme’s significant biological activity and its potential for future applications in the treatment of cancer-related diseases (Figure 5a). Li et al. [113] designed and synthesized a carbon-doped nanozyme restricted in a nitrogen-doped carbon framework through pyrolysis of a ZIF-8 precursor filled with glucose (G@ZIF-8). The CD nanozyme demonstrated robust peroxidase-like activity, converting H_2_O_2_ into superoxide radicals and oxidizing TMB molecules into blue oxTMB during the chromogenic reaction. To explore the role of the carbon host and CD guest in the catalytic activity of CDs@NC-3, the authors synthesized the NC host and CDs separately and investigated their catalytic activities. The results indicated that the synergetic effect was not the dominate factor in the improvement of catalytic activity and instead was the confinement effect that played a pivotal role in the improvement of catalytic activity. Notably, the saliva of early gastric cancer patients was found to contain elevated levels of D-proline (D-Pro) and D-alanine (D-Ala), while D-amino acid oxidase exhibited chiral-specific catalytic properties in alkaline and neutral conditions, selectively catalyzing D-Pro and D-Ala in these patients. This specific catalysis involved the conversion of D-Pro and D-Ala into H_2_O_2_, followed by TMB oxidation based on the H_2_O_2_-related catalytic activity of CDs@NC, enabling the detection of D-Pro and D-Ala (Figure 5b). Wang et al. [65] utilized hydrothermal synthesis to produce a nitrogen-doped CD nanozyme with oxidase-like activity. This nanozyme was created by using tea polyphenol and ethylenediamine as precursor. Compared to tea polyphenol alone, the nanozyme displayed significantly enhanced antimicrobial properties. The introduction of nitrogen doping resulted in the formation of surface-active sites such as pyrrolyl nitrogen and graphitic nitrogen structures, allowing the enzyme to alter its electronic energy level and boost its catalytic activity. The enzyme catalyzed O_2_ to generate superoxide radicals under light radiation. These radicals subsequently oxidated TMB to oxTMB. The nanozyme effectively penetrated the bacterial cell membranes, interacting with intracellular proteins, lipids and nucleic acids, disrupted of cellular structure and function by generating superoxide radicals, thereby inhibiting or eradicating the bacteria. Moreover, the antimicrobial efficacy of the nanozyme was significantly higher under light exposure compared to dark conditions. Furthermore, the antibacterial activity exhibited a synergistic relationship between the concentration of the nanozyme and the duration of light exposure. As the concentration and duration of light increased, this synergistic effect resulted in improved antibacterial efficiency (Figure 5c).

### 3.3. Metal-Doped CDs Nanozymes

**Figure 6:**
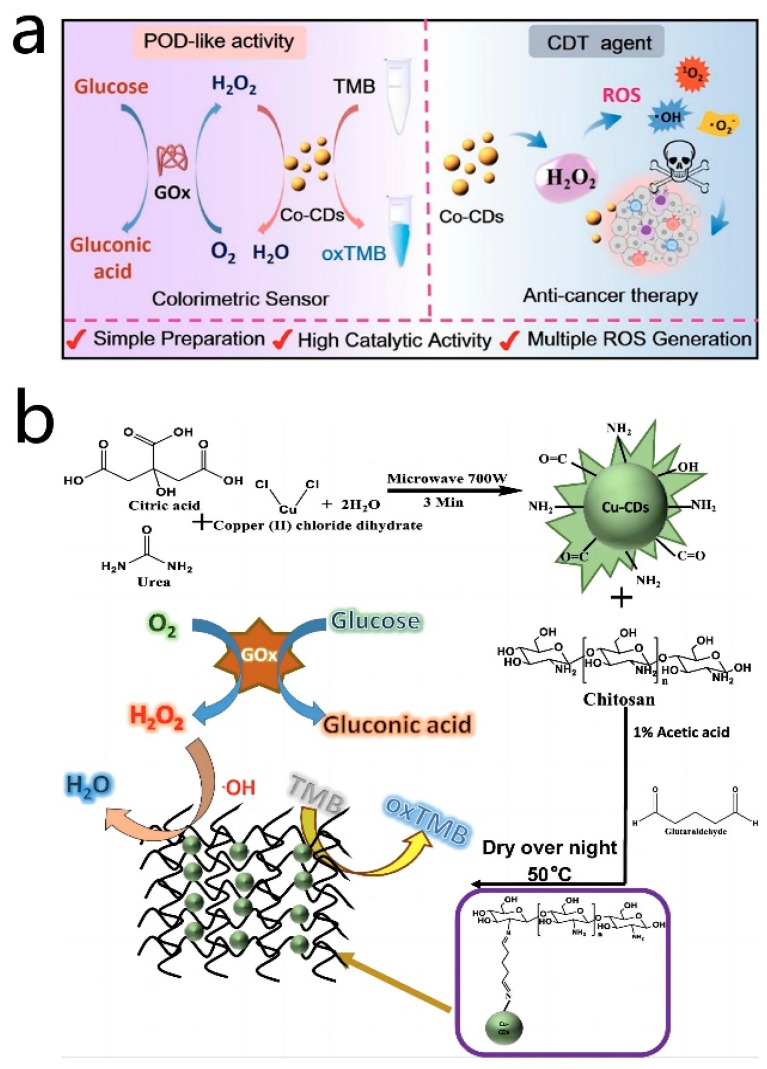
(**a**) Illustration of the Co-CD nanozyme for colorimetric determination of glucose and anticancer cell effect. Reprinted with permission from ref. [69]. Copyright 2022, American Chemical Society. (**b**) Schematic representation for the synthesis of Cu-CDs/chitosan film and the detection of H_2_O_2_ and glucose. Reprinted with permission from ref. [116]. Copyright 2022, Srikrishna Tummala et al.

Doping metal atoms into CD nanozymes can significantly enhance their optical properties and catalytic abilities, owing to the unique structures of the metal atoms and other associated factors. Different metals utilized for doping can yield distinct effects on the properties and catalytic performances of CD nanozymes [114,115]. For instance, Zhuo et al. [68] developed a rapid and efficient colorimetric assay for the measurement of ascorbic acid using manganese-doped CD nanozymes. The Mn-CD nanozymes exhibit oxidase-like activity, whereby the dissolved oxygen could interact with the active sites on the surface of Mn-CD, leading to the generation of ROS. These ROS then catalyzed the conversion of TMB to blue oxTMB. Upon the addition of ascorbic acid (AA) to the catalytic system, AA reacted with the oxTMB, reducing it back to the colorless TMB. This catalytic system demonstrated high selectivity for AA and offered a significant advantage in detecting AA in real samples. Moreover, Lu et al. [69] synthesized Co-doped carbon nanozymes with strong peroxidase-like activity through hydrothermal synthesis using vitamin B_12_ and citric acid as precursors. In this process, cobalt ions were present in the form of Co(II) and Co(III). Co(II) ions demonstrated excellent Fenton reactivity, enabling the decomposition of H_2_O_2_ and the production of hydroxyl radicals (•OH), ultimately converting TMB to oxTMB. Simultaneously, in the presence of H_2_O_2_, Co-CDs could be oxidized, leading to the generation of peroxyl radicals following the Russell mechanism, which subsequently combined to form singlet oxygen (^1^O_2_). Moreover, Co-CDs exhibited the capability to interact with glucose oxidase (GOx), which oxidized glucose into H_2_O_2_ and gluconic acid. Subsequently, Co-CDs leverage this H_2_O_2_ to catalyze the oxidation of TMB, resulting in the formation of ox-TMB with distinct absorption peaks at 652 nm. The good linear correlation between the absorbance of oxTMB and the glucose concentration allowed for the precise and quantitative detection of glucose. Additionally, Tummala et al. [116] utilized the microwave radiation method to prepare copper-doped CD nanozymes, which were subsequently doped into chitosan membranes through a cross-linking reaction to create a composite material. The cross-linking reaction between amino groups and Cu-CDs played a crucial role in enhancing the stability of the membranes and preventing particle aggregation during the analysis process. The resulting composite exhibited peroxidase-like activity, facilitating the catalysis of the H_2_O_2_-mediated TMB oxidation reaction. This catalytic activity was particularly effective under acidic conditions, leading to the generation of blue oxTMB. A standard curve established by the colorimetric detection of TMB enabled the quantitative detection of H_2_O_2_ and glucose concentrations. This method not only allowed for the colorimetric detection of H_2_O_2_ and glucose but also mitigated issues related to particle aggregation arising from the presence of chitosan membranes. Ultimately, this approach proved valuable in the analysis of glucose sugars in human serum (Figure 6b). In addition, there were a variety of doping elements, such as Fe [67,79,117,118,119], Sc [120], Ce [121] and Mo [70]. Such metal-doped CD nanozymes have been used in analytical and biomedical applications, with promising application prospects.

### 3.4. Codoped CD Nanozymes

**Figure 7:**
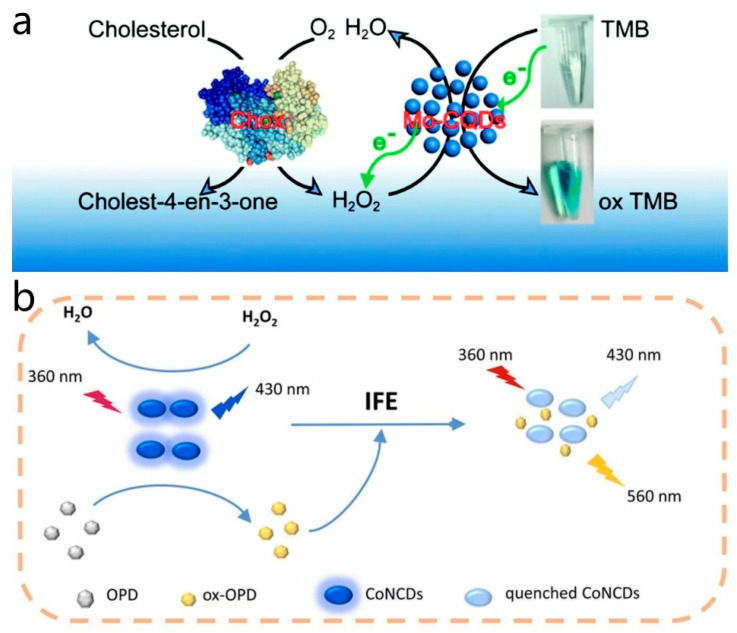
(**a**) Schematic diagram of cholesterol detection by Mo, S-CDs and cholesterol oxidase. Reprinted with permission from ref. [71]. Copyright 2019, Royal Society of Chemistry. (**b**) Ratio fluorescence multimodal analysis strategies for H_2_O_2_ detection. Reprinted with permission from ref. [77]. Copyright 2021, Elsevier.

Codoped CD nanozymes, which are prepared by doping two or more elements to enhance the properties as well as the catalytic ability of CD nanozymes, have been a focus of research in recent years. Zhao et al. [71] synthesized a novel Mo and S codoped CD nanozyme with strong peroxidase-like activity using a hydrothermal synthesis method. The codoping of Mo and S significantly increased the production yield of the fabricated CD nanozyme. The functional groups on the surface of the enzyme could interact with the amino groups of TMB, facilitating the transfer of electron pairs from TMB to the enzyme. This enhanced the electron density and mobility of the enzyme, thereby boosting its catalytic activity. Due to its peroxidase-like activity, this enzyme can catalyze the color reaction of TMB to oxTMB in the presence of H_2_O_2_. When combined with the action of cholesterol oxidase (Chox), which generates H_2_O_2_ through the oxidation of cholesterol, the codoped CD nanozyme catalyzes the reaction between H_2_O_2_ and TMB, resulting in a color change. This color change allows for the visual detection of cholesterol concentration, enabling highly sensitive cholesterol testing (Figure 7a). Su et al. [77] developed and prepared a cobalt and nitrogen codoped CD nanozyme, which exhibited excellent peroxidase-like activity and fluorescence characteristics in neutral and alkaline environments. The Co(II) center in Co, N-CDs served as the active site that interacted with H_2_O_2_. In the presence of H_2_O_2_, the Co(II) center was oxidized to Co(III), initiating the catalytic cycle. Under the influence of the Co(II) center, o-phenylenediamine (OPD) molecules were oxidized to the corresponding product oxOPD, involving electron transfer where the Co(II) center accepts electrons from OPD while transferring electrons to H_2_O_2_, facilitating its decomposition into reactive hydroxyl radicals (•OH). As OPD was oxidized and oxOPD was formed, the Co(III) center was reduced back to Co(II), preparing for the next catalytic cycle. Co, N-CDs exhibited higher catalytic activity under neutral to alkaline conditions but lower activity under acidic conditions, likely due to the charge state of the Co, N-CDs surface. In neutral to alkaline environments, the negatively charged surface of Co, N-CDs favors electrostatic adsorption with the positively charged OPD substrate, promoting catalytic reactions. The enzyme displayed selectivity towards the OPD substrate, possibly attributed to the surface properties of Co, N-CDs and the electronic structure of the Co(II) center, enhancing its affinity and catalytic efficiency towards OPD. By catalyzing the oxidation of OPD in the presence of H_2_O_2_, the enzyme underwent fluorescence quenching, allowing for the construction of the colorimetric and ratiometric fluorescence method for determining H_2_O_2_ based on the absorbance of ox-OPD at 420 nm and the fluorescence intensity ratio of ox-OPD to CoNCDs (I_560_/I_430_). The colorimetric and fluorescent detection method was based on the absorbance and fluorescence intensity ratio of the corresponding substances to determine the H_2_O_2_ content. Utilizing this H_2_O_2_ measurement method, in conjunction with cholesterol oxidase or xanthine oxidase catalyzing the production of H_2_O_2_ from cholesterol or xanthine, enabled the detection of cholesterol and xanthine in human serum samples. This method offered high sensitivity in detecting cholesterol or xanthine and produced multiple fluorescence signals, reducing potential interference during the detection process and increasing the precision of the method (Figure 7b). In a similar vein, Dong et al. [78] synthesized cobalt and nitrogen codoped CD nanozymes. Unlike other enzymes, this enzyme exhibited peroxidase-like activity. The Co doping created the active center, which interacted with the surface or internal structure of the CDs, providing an appropriate electron environment for catalyzing oxygen activation. The presence of cobalt ions enhanced the affinity of Co, N-CDs for oxygen by providing unpaired electrons, thereby promoting oxygen activation. Initially, oxygen (O_2_) molecules were adsorbed by the active center of Co, N-CDs. The interaction between oxygen molecules and cobalt ions altered the electronic structure of the oxygen molecules, reducing their oxidation potential and facilitating the release of reactive oxygen species. Facilitated by the active center, Co, N-CDs convert oxygen molecules into singlet oxygen (^1^O_2_) through an electron transfer process. The reaction between ^1^O_2_ and extracellular DNA (eDNA) in bacterial biofilms leads to DNA strand breakage, disrupting the structure and function of the biofilm, rendering bacteria vulnerable. Furthermore, ^1^O_2_ not only damaged the biofilm but also reacted directly with bacterial cell membranes and internal components, causing oxidative damage, compromising the integrity of the cell membrane and ultimately resulting in bacterial death. This enzyme exhibited broad-spectrum antibiofilm activity against both Gram-positive and Gram-negative bacteria.

### 3.5. Undoped CD Nanozymes

Undoped CD nanozymes are prepared by using a single carbon source as a precursor substance, and the prepared CDs are free from elemental doping and have certain enzyme-like catalytic activities. Lin et al. [87] prepared undoped CD nanozymes by hydrothermal synthesis, using citric acid as a single carbon source. These nanozymes exhibited cysteine oxidase-like activity, catalyzing the oxidation of cysteine to cysteamine and H_2_O_2_. Compared to natural cysteine oxidase, the CD nanozymes demonstrated higher catalytic activity. The active sites on the enzyme surface catalyzed the further decomposition of H_2_O_2_ to generate highly reactive hydroxyl radicals (•OH). The hydroxyl radicals (•OH) possessed strong oxidizing properties, facilitating the oxidation of terephthalic acid (TA) to produce highly fluorescent hydroxyterephthalic acid (TA-OH). The TA-OH emitted strong fluorescence at specific wavelengths, allowing for indirect quantification of cysteine concentration by measuring the fluorescence intensity. This method offered low detection limits, high selectivity and excellent stability, presenting a novel tool for biomedical analysis and clinical diagnosis (Figure 2a). Geng et al. [122] developed undoped CD nanozymes with highly efficient superoxide dismutase-like enzyme activity. The catalytic active center of this enzyme contained specific chemical groups that can interact with •O^2−^ and H_2_O_2_, providing an electron to •O^2−^ to reduce it to oxygen. Simultaneously, other groups on the enzyme could bind to H_2_O_2_, further converting it into water and oxygen. In addition to SOD-like enzyme activity, this enzyme also exhibited antioxidant properties by decomposing H_2_O_2_ into water and oxygen, thereby helping to reduce the toxicity of reactive oxygen species (ROS). By scavenging •O^2−^ around mitochondria, this enzyme protected mitochondrial function, inhibiting cell apoptosis and reducing levels of inflammatory cytokines, effectively mitigating oxidative stress and inflammatory responses within the body and protecting cellular damage. Through regulating the hepatic inflammatory network and suppressing apoptosis, it achieved the therapeutic effect of treating liver ischemia/reperfusion injury.

## 4. Summary and Prospects

The absorption of light energy in the catalytic process of enzymes may result in a modification of the internal electronic state of the enzyme molecule, consequently influencing its activity. Photoluminescence is the phenomenon where a substance absorbs photons and emits photons. CD nanozymes, distinguished by their unique photoluminescent attributes, can exhibit catalytic behavior comparable to natural enzymes under light excitation, while they do not display thermocatalytic activity in the absence of light. By introducing new functional groups or structures onto the surface of CDs, the dots’ activity can be altered. For example, by incorporating functional groups like amino, carboxyl and hydroxyl onto the dot’s surface, these can attach to the dots in a covalent or non-covalent manner, thereby imparting the dots with specific bioactivity or catalytic property. Furthermore, surface functionalization has repercussions on the optical properties of CDs. The addition of various fluorescent molecules or metal nanoparticles can alter the luminescence wavelength and quantum yield of CDs, alongside enhancing water solubility and biocompatibility, rendering them more suitable as carriers for bioimaging and drug-delivery purposes.

Graphene quantum dots (GQDs) and CDs are nanomaterials that demonstrate enzyme-like catalytic activity. GQDs exhibit peroxidase-like activity, particularly GQDs possessing a higher oxygen content, leading to increased peroxidase-like activity with lower Km for the substrate H_2_O_2_. This denotes a higher catalytic efficiency. Conversely, CDs display SOD-like activity, enabling them to scavenge superoxide anions. The catalytic activity of CDs is intricately linked to the presence of surface functional groups such as carbonyl and hydroxyl. These functional groups interact with superoxide anions via hydrogen bonding, capturing electrons to yield oxygen and reduced-state CDs, manifesting their SOD-like activity. Although both GQDs and CDs exhibit enzyme-like properties, variations exist in their catalytic mechanisms and active sites. GQDs’ peroxidase-like activity is predominantly associated with their oxygen-containing functional groups, whereas the SOD-like activity of CDs is contingent upon the abundance of functional groups on their surfaces and specific chemical alterations.

The study of CD nanozymes is still in its infancy compared with quantum dots and other carbon materials. Although there have been numerous descriptions of the mechanism of action of CD nanozymes, the detailed catalytic function mechanism remains not fully understood. Further research is required to comprehend the relationship between their structure and function and to achieve precise development and application of their functions. Currently, limitations still exist in the characterization of CDs, necessitating the development of more effective characterization techniques to identify the luminescence center or mechanism. Despite the relatively simple preparation of CDs, the urgent challenge of the large-scale production of high-quality and enzyme-specific CDs remains.

To accurately classify the various types of CD nanozymes, it is essential to establish a more systematic classification system. Currently, most of the discovered CD nanozymes predominantly exhibit oxidoreductase activity. Expanding research efforts to develop CD nanozymes with a wider range of catalytic types is crucial to broaden their application potential. Despite demonstrating lower toxicity in vitro compared to other materials, it is imperative to consider the biological safety of CD nanozymes during their application. Detailed investigations into their pharmacokinetics and other aspects are necessary to ensure the safe utilization of CD nanozymes in body treatments and other applications.

CD nanozymes are relatively simple to prepare and offer strong enzyme-like activity and optical properties, making them valuable for applications in sensing, medicine and therapy. The introduction of doping elements can further enhance the properties and catalytic activity of CD nanozymes. Due to their superior biocompatibility, stability, lower cost compared to other nanomaterials and relatively low toxicity, CD nanozymes hold significant promise for advancements in biomedicine and therapy. This paper provides a comprehensive overview of recent research progress on CD nanozymes, detailing common preparation methods and discussing their diverse applications based on doping types. With the continuous progress of research on CD nanoenzymes, their potential for widespread application is expected to be further expanded.

## Figures and Tables

**Table 1:** Preparation materials and enzyme-like activities of various CD nanozymes under different preparation methods.

Preparation Method	CDs	Precursor Material	Reaction Condition	Enzyme-like Activity	Applications	Ref.
Hydrothermal synthesis	CDs	Citric acid (CA), Ehylenediamine (EDA)	200 °C, 8 h	OXD-like, POD-like	Photodynamic therapy	[62]
	N-CDs	Valine	200 °C, 12 h	POD-like	/	[63]
	N-CDs	CA, EDA	200 °C, 1 h	POD-like	Optical probe	[64]
	N-CDs	Tea polyphenols, EDA	200 °C, 10 h	OXD-like	Antibacterial	[65]
	N-CDs	Tartaric acid, 3-aminophenol	160 °C, 12 h	OXD-like	Tumor therapy	[66]
	Fe-CDs	NaFeEDTA	350 °C, 2 h	POD-like	Antibacterial	[67]
	Mn-CDs	MnCl_2_·4H_2_O, CA, EDA	180 °C, 10 h	OXD-like	Colorimetric detection	[68]
	Co-CDs	Vitamin B_12_(VB_12_), CA	180 °C, 3 h	POD-like	Biosensor and Anticancer	[69]
	Mo-CDs	(NH_4_)_6_Mo_7_O_24_·4H_2_O, ascorbic acid(AA)	200 °C, 12 h	POD-like	Colorimetric sensing	[70]
	Mo, S-CDs	Ethanol, MoS_2_	200 °C, 12 h	POD-like	Colorimetric sensing	[71]
	Cu, Cl-CDs	CA, CuCl_2_·2H_2_O, choline chloride, etc.	180 °C, 6 h	OXD-like, POD-like	Colorimetric detection	[72]
	Cu, I-CDs	CuCl_2_, EDA, 3-Iodo-L-tyrosine	180 °C, 8 h	POD-like	Antifungal	[73]
	N, Cl-CDs	3-Iodo-L-tyrosine, Iopromide3,5-Diiodo-DL-tyrosine	180 °C, 10 h	POD-like	Antifungal	[74]
	Fe, N-CDs	FeCl_3_·6H_2_O, Na_2_EDTA	200 °C, 10 h	POD-like	Biosensor detection	[75]
	Fe, N-CDs	Fe(NO_3_)_3_·9H_2_O, Diethylenetriamine pentaacetic acid	180 °C, 10 h	POD-like	Colorimetric detection	[76]
	Fe, N-CDs	CA, FeSO_4_·7H_2_O, p-phenylenediamine	180 °C, 8 h	POD-like	Antibacterial	[52]
	Co, N-CDs	Co(NO_3_)_2_·6H_2_O, Na_2_EDTA	300 °C, 2 h	POD-like	Biosensing and bioimaging	[77]
	Co, N-CDs	EDTA–Na, Co(NO_3_)_2_·6H_2_O	300 °C, 2.5 h	OXD-like	Antibacterial treatment	[78]
	Fe, N, S-CDs	Sunset yellow, FeCl_3_	200 °C, 3 h	POD-like	Colorimetric detection	[79]
Microwave radiation	CDs	CA, L-histidine	700 W, 2 min	OXD-like	Photodynamic therapy	[37]
	Fe-CDs	CA, FeCl_2_·4H_2_O	700 W, 14 min	POD-like	Colorimetric detection	[51]
	Fe-CDs	Bougainvillea plant leaves, FeCl_3_	630 W	OXD-like, POD-like, SOD-like, catalase-like (CAT-like), glutathione peroxidase-like (GPx-like), thiol peroxidase-like (TPx-like)	Tumor therapy	[80]
	Cu-CDs	Cu(CH_3_COO)_2_·H_2_O, urea, CA	220 °C, 10 min	POD-like	Colorimetric detection	[81]
	S, N-CDs	CA, thiourea	\	OXD-like	Antibacterial	[82]

## Data Availability

Not applicable.
